# Molecular Markers Guiding Thyroid Cancer Management

**DOI:** 10.3390/cancers12082164

**Published:** 2020-08-04

**Authors:** Carolina Nylén, Robert Mechera, Isabella Maréchal-Ross, Venessa Tsang, Angela Chou, Anthony J. Gill, Roderick J. Clifton-Bligh, Bruce G. Robinson, Mark S. Sywak, Stan B. Sidhu, Anthony R. Glover

**Affiliations:** 1 Endocrine Surgical Unit, Royal North Shore Hospital, Northern Sydney Local Health District, St. Leonards, NSW 2065, Australia; carolina.nylen@ki.se (C.N.); Robert.mechera@gmail.com (R.M.); marksywak@nebsc.com.au (M.S.S.); stansidhu@nebsc.com.au (S.B.S.); 2 Department of Molecular Medicine and Surgery, Karolinska Institutet, Karolinska University Hospital, Solna L1:00, 171 76 Stockholm, Sweden; 3 Department of Visceral Surgery, Clarunis University Hospital Basel, Spitalstrasse 21, 4031 Basel, Switzerland; 4 Northern Clinical School, Sydney Medical School, Faculty of Medicine and Health, University of Sydney, Sydney, NSW 2006, Australia; imar3287@uni.sydney.edu.au (I.M.-R.); venessa.tsang@sydney.edu.au (V.T.); angelashihyuan.chou@health.nsw.gov.au (A.C.); affgill@med.usyd.edu.au (A.J.G.); roderick.clifton-bligh@sydney.edu.au (R.J.C.-B.); bruce.robinson@sydney.edu.au (B.G.R.); 5 Department of Endocrinology, Royal North Shore Hospital, University of Sydney, St. Leonards, NSW 2065, Australia; 6 NSW Health Pathology, Department of Anatomical Pathology, Royal North Shore Hospital, University of Sydney, St. Leonards, NSW 2065, Australia; 7 Cancer Genetics Unit, Kolling Institute, Sydney, NSW 2010, Australia; 8 The Kinghorn Cancer Centre, Garvan Institute of Medical Research, Faculty of Medicine, St. Vincent’s Clinical School, University of New South Wales Sydney, Sydney, NSW 2010, Australia

**Keywords:** Genomics, molecular testing, thyroid cancer, surgery, indeterminate nodules, tyrosine kinase inhibitors, targeted therapy, personalized medicine

## Abstract

The incidence of thyroid cancer is rapidly increasing, mostly due to the overdiagnosis and overtreatment of differentiated thyroid cancer (TC). The increasing use of potent preclinical models, high throughput molecular technologies, and gene expression microarrays have provided a deeper understanding of molecular characteristics in cancer. Hence, molecular markers have become a potent tool also in TC management to distinguish benign from malignant lesions, predict aggressive biology, prognosis, recurrence, as well as for identification of novel therapeutic targets. In differentiated TC, molecular markers are mainly used as an adjunct to guide management of indeterminate nodules on fine needle aspiration biopsies. In contrast, in advanced thyroid cancer, molecular markers enable targeted treatments of affected signalling pathways. Identification of the driver mutation of targetable kinases in advanced TC can select treatment with mutation targeted tyrosine kinase inhibitors (TKI) to slow growth and reverse adverse effects of the mutations, when traditional treatments fail. This review will outline the molecular landscape and discuss the impact of molecular markers on diagnosis, surveillance and treatment of differentiated, poorly differentiated and anaplastic follicular TC.

## 1. Introduction

According to the Global Cancer Observatory GLOBOCAN 2018 database, thyroid cancer (TC) accounts for 3.1% of all new cancer diagnoses worldwide and, therefore, ranks in 9th position with regard to incidence [1,2]. Moreover, with an estimated annual 41,000 deaths, mortality rates (0.4–0.5%) remain comparably stable and low [1,3]. However, it is one of the fastest growing cancer entities worldwide and its incidence in the United States has nearly tripled over the last 30 years from 4.9 to 14.3 per 100,000 people [3,4,5]. Many reasons for this epidemiological development have been discussed [2,6,7,8]. While some authors also suggest a contributing true increase of incidence [9], the main reason for the increase seems to be a consequence of oversurveillance and overdiagnosis of small differentiated tumours [3]. This can be attributed to significant improvements of diagnostic techniques, particularly higher sensitivity of ultrasounds (US), US-guided fine needle aspiration (FNA) and computed tomography [7,10]. The resulting overtreatment or continuous surveillance for clinically insignificant findings not only influences incidence but is also a significant factor in times where modern healthcare systems become under significant cost pressure [11,12].

Therefore, it is crucial to identify adjuncts such as molecular markers, which can distinguish between benign and malignant tumours, predict aggressive biology, prognosis, recurrence and efficacy of treatment as well as represent possible novel therapeutic targets. The development of potent preclinical models, high throughput molecular technologies such as next-generation sequencing (NGS) and gene expression microarrays have provided a deeper understanding of genetic alterations and molecular characteristics in cancer [13]. This offers the potential for development of personalized cancer treatment where patients are treated according to their individual tumour characteristics, with potential to improve patient’s outcome, rather than to a “one-fits-all” strategy [14]. The increasing importance of molecular markers in diagnostic and treatment strategies of TC was also further acknowledged by the latest American Thyroid Association (ATA) guidelines as well as American Association of Endocrine Surgeons guidelines [15,16,17].

TC subtypes derived from follicular cells can be histologically divided into differentiated (DTC), poorly differentiated (PDTC), and anaplastic thyroid cancer (ATC) [18]. However, since the discovery of the oncogenic role of the *BRAF^V600E^* mutation in 2003, various molecular and genetic markers in TC have been identified, and added an additional layer to the classification of TC [19,20,21]. Although the total mutational burden (TMB) is lower than in most other cancer entities, the increasing importance of molecular alterations was underlined by The Cancer Genome Atlas (TCGA), which identified genetic alterations in 97% of the studied tumours [20,22]. While the TCGA analysis only included papillary thyroid cancer (PTC), subsequent studies have shown several markers and their combination play a significant role in the development of other TC subtypes as well as the progression and dedifferentiation to more advanced cancer subtypes [23,24,25]. An example of evolving biomarkers for diagnosis and prognosis in TC are MicroRNAs (miRNA). By regulating target genes of various signalling pathways including gene expression of known oncogenes and tumour suppressor genes, they play a crucial role for cell differentiation, migration, invasion and even epithelial-to-mesenchymal transition (EMT) [26,27,28]. Furthermore, different and distinct miRNA profiles were identified in different TC subtypes [28,29,30,31,32,33].

In this review, we will outline the molecular landscape of follicular cell derived TC and discuss the impact of molecular markers on diagnosis, surveillance, and treatment of DTC, as well as PDTC and ATC. Moreover, we will give an outlook on the emergence of novel molecular characteristics and techniques, which have potential to guide decision making in TC management in the future leading to personalisation of TC care and avoiding overtreatment in low risk cancers.

## 2. The Molecular Landscape of Follicular Cell Derived Thyroid Cancer and Oncogenesis

**Figure 1:**
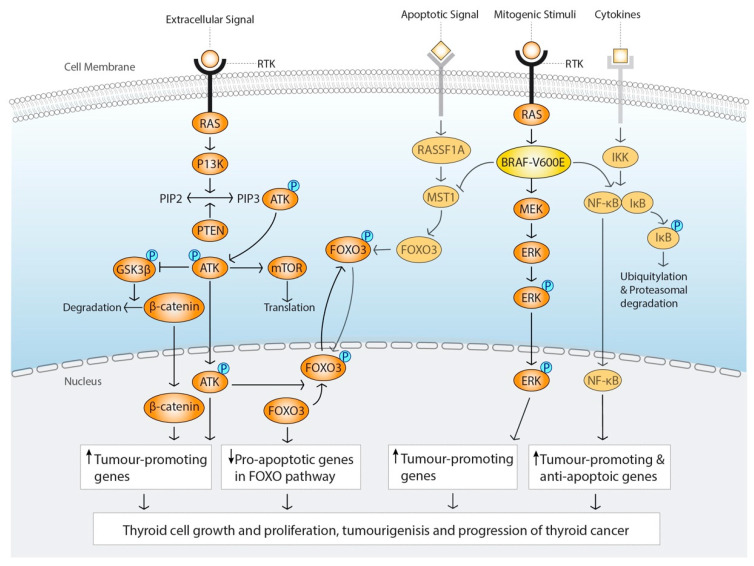
Molecular landscape in thyroid cancer.

The understanding of the molecular landscape of follicular cell derived TC has improved vastly with the publications of the genomic studies of TC, which has enabled promising new targets of treatment. The mutations identified in the TCGA show distinct patterns mostly affecting two signalling pathways involved in thyroid growth and proliferation, the mitogen-activating protein kinase (MAPK), and the Phosphatidylinositol-3-kinase (PI3K)/AKT-pathways [34] (Figure 1). Genetic and epigenetic dysfunctions of kinases and transcription factors related to these pathways lead to constitutive activation that initiate and aggravate the tumorigenesis and progression of TC. In this section, we will describe the main identified molecular alterations in follicular-derived TC.

### 2.1. The MAPK-Signalling Pathway

The MAPK-signalling pathway is crucial for growth and proliferation of the cell (Figure 1). The signalling cascade is initiated on the cell surface by a “mitogen” stimuli, a growth factor, that binds to the tyrosine kinase receptor (RTK) and ultimately results in the phosphorylation and activation of Mitogen-Activated Protein Kinase Kinase (MAP2K), which is also known as MEK, and the downstream extra cellular signal-regulated-kinase (ERK). Phosphorylation of ERK leads to activation of transcription factors targeting genes to induce cell cycle entry and to suppress negative regulators of cell cycle [35]. The MAPK-signalling pathway is delicately regulated in several steps by enzymes and factors, to which genetic and epigenetic dysregulations can lead to a constitutively active pathway, an uncontrolled growth and proliferation of the cell, hallmarks of oncogenesis [36]. Mutations of key regulators of the MAPK-signalling pathway are present in about 70% of all PTC [37] and constitutive MAPK activation leads to dedifferentiation of PTC in preclinical models [38].

This illustration summarizes the two main affected signalling pathways in thyroid cancer (TC); Phosphatidylinositol-3-kinase (PI3K)-AKT and mitogen-activating protein kinase (MAPK) signalling pathway. Mutations or genetic rearrangements of targets in the respective pathway lead to constitutive activation that ultimately leads to translocation of transcription factors to the nucleus upregulating transcription of genes promoting tumourigenesis and progression.

A point mutation at the loci T1799A in codon 600 in the B-Raf proto-oncogene, *BRAF*, a gene coding for a key serine/threonine kinase in the MAPK signalling cascade, leads to a *BRAF*^V600E^ mutant protein first described in TC by Cohen et al. in 2003 [19]. *BRAF*^V600E^ mutation leads to a constitutive active BRAF kinase independent of its upstream target, *RAS*, ultimately leading to increased ERK1/2 activation [39]. The *BRAF*^V600E^ kinase domain is about 500 fold more active than the wild type BRAF kinase [40] and its mutation is regarded to be one of the fundamental initiating events in the tumorigenesis and progression of PTC [41].

In addition to activating the downstream targets in the MAPK-signalling cascade, *BRAF*^V600E^ mutation is also capable of activating the nuclear factor kappa beta (NFκB) pathway [42], which is important for the inflammatory response and activated in oncogenesis. *BRAF*^V600E^ mutation can also inhibit the class O of forkhead box transcription factors (FOXO)-pathway through macrophage stimulating 1 (MST1), leading to inhibition of proapoptotic genes [43].

The discovery of telomerase reverse transcriptase (TERT) promoter mutations in TC in 2013 [44], represents an important event in the TC field and has gained much attention since in its role in modifying the effect of driver mutations such as *BRAF*. The proposed mechanism of *TERT* promoter mutation is to generate de novo binding elements for the ETS-transcription family of transcription factors such as GABPA, which leads to increased telomerase expression and immortalisation [45]. ETS-transcription factors are activated by MAPK signalling, and this process enhances the effect of the MAPK signalling further, and indeed, co-existent mutations of *BRAF*^V600E^ and *TERT* are associated with more aggressive forms of PTC [46].

### 2.2. PI3K/AKT Signalling Pathway

The PI3K/AKT signalling pathway is involved in the regulation of apoptosis, proliferation, cell cycle progression, angiogenesis, cytoskeleton integrity, and energy metabolism, and ultimately leads to AKT and mTOR activation [47] (Figure 1). One of the most commonly mutated targets in the PI3K/AKT cascade is *RAS*. RAS, a small G protein anchored to the inner membrane of the cell, is the upstream kinase of BRAF kinase and active when bound to GTP. *RAS* mutation downregulates the activity of GTPase, the enzyme that hydrolyses GTP to GDP, and the mutation leaves RAS in a constitutively active GTP-bound state. Due to its location in the cell, adjacent to the membrane bound tyrosine kinase receptor, RAS mutations can activate both MAPK- and PI3K/AKT pathways. However, it seems like *RAS* mutations preferable activate the PI3K-AKT pathway in thyroid oncogenesis [48]. There are three isoforms of RAS-NRAS, HRAS and KRAS, where *NRAS* is the most commonly mutated in human TC [49]. *RAS* mutation is thought to be a premalignant mutation, and that additional mutations are needed to set off carcinogenesis [49]. Indeed, in a *KRAS* transgenic mouse model, deletion of a tumour suppressor gene, PTEN, was needed for development of follicular thyroid cancer (FTC) [50]. Mutations or deletions of PTEN activates PI3K-AKT pathway, which is the genetic basis for follicular thyroid cell tumorigenesis in Cowden’s disease [51]. Mutations in PI3K3CA gene which encodes for the p110alpha catalytic subunit of PI3K is also common in follicular thyroid cancer (FTC), but also poorly differentiated thyroid cancer (PDTC) and anaplastic thyroid cancer (ATC) [48].

### 2.3. Gene Translocations

Gene translocations can lead to oncogenetic chromosomal rearrangements. The most common group of translocation in TC is known as *RET/PTC* and was first described in PTC by Fusco et al. 1987 [52]. It is important to note that *RET/PTC* does not refer to a single translocation, but to a group of different translocations involving *RET* and several other genes. For example, the most common translocation known as *RET/PTC1* and *RET/PTC3* actually represent translocations between the *RET* gene and *CCDC6* (*RET-CCDC6*) and the *RET* gene and *NCOA4* (*RET-NCOA4*), respectively [53]. *RET/PTC* rearrangements occurs as a consequence of genetic recombination between the 3´tyrosine kinase portion of RET and the 5´portion of a partner gene due to spatial proximity in the nucleus [54]. *RET* is a classical proto-onco gene, a gene that when activated by mutation on rearrangement can contribute to cancer. The *RET/PTC* rearrangement encodes for a fusion oncogene containing a receptor tyrosine kinase (RTK) that is ligand independent, enabling constitutive activation of both MAPK and the PI3K-AKT pathways. The *PAX8*/*PPARG, NTRK1, and -3* and *ALK* are other prominent recombinant oncogenes [55], where the fusion of the oncogenes alters the transcription of the target genes, predisposing for oncogenesis in TC.

### 2.4. Gene Amplification and Copy Number Alterations

Oncogenic gene amplifications are genetic mechanisms in thyroid oncogenesis, commonly occurring with RTKs (receptor tyrosine kinases) and genes encoding targets in the PI3K-AKT pathways. Genetic copy number alterations (CNA) are more common in ATC suggesting mechanism of progression and aggressiveness [25]. CNA through genetic amplification or chromosomal instability and aneuploidy (extra chromosome) leads to increased protein expression and hence activation of downstream signalling pathways. Gains of genes encoding RTKs ultimately increase phosphorylation and hence activation of both AKT and ERK [48].

### 2.5. Epigenetic Regulation

“Epi” comes from the Greek prefix meaning “on top of”, which refers to a regulative mechanism “on top of the DNA sequence”. Epigenetic modifications of the genome and gene expression include histone modifications, DNA methylation and microRNAs (miRNA). These epigenetic marks influence the central dogma of DNA transcription, RNA (mRNA) translation and protein synthesis in the cell. Epigenetic modifications are common in human cancer and are also present in TC [56]. DNA methylation of promotor regions often lead to suppression of gene transcription. In array-based genome-wide DNA methylation studies of PTC, the first performed by Hou et al. [57] shows that, in similarity to sequencing studies revealing low mutation frequency in PTC, there is also low frequency of DNA methylation alteration. In contrast, ATC exhibits a high frequency of DNA methylation alterations, 10-fold higher than PTC [58]. One confounding factor in performing DNA methylation studies on ATC is however the infiltration of immune cells making it possible that part of the hypermethylation may come from these cells and not the TC itself [56]. Some specific targets that often have been shown to be hypermethylated in TCs is *PTEN*, *RASSF1A*, *CDKN2A* or *P16INK4A* and *DAPK* [56].

Histones are the scaffold proteins that DNA is tightly winded around and function to condense and package DNA in the nucleus. Histones have molecules attached to them that when modified changes the chromatin structure and the availability of the DNA sequence to transcription [59]. There are several different known histone modifications and enzymes responsible for these modifications. Most of the histone modifications identified are also in the more advanced TCs, and the most studied is the histone deacetylase (HDAC) [60].

He et al. [61] were the first to acknowledge the involvement of miRNA in PTC. MiRNA are noncoding small RNAs that in simplified terms recognise a target messenger RNA (mRNA) and repress translation of the mRNA into protein, and will be discussed further below. In addition to miRNA, the involvement of multiple long noncoding RNAs (lncRNA) in TC have recently been established by several researchers [62,63]. Dysregulated lncRNA are involved in the epigenetic regulation of gene transcription and implicated in tumour suppression and oncogenic functions [64]

### 2.6. The Progressive Oncogenesis Model

**Figure 2:**
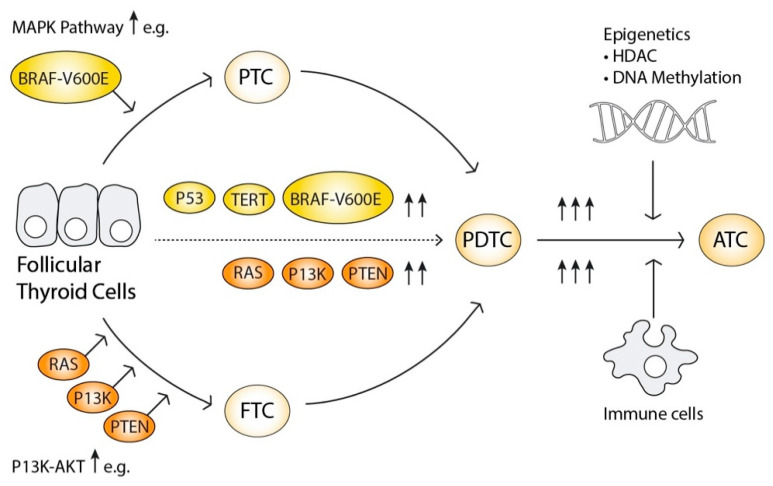
Illustration of the oncogenesis mode.

The currently accepted model of TC oncogenesis is the multistep oncogenesis model, that suggests that mature follicular cells can transform into well differentiated TC cells and then progress into undifferentiated TC cell [65] (Figure 2). The clinical findings that well differentiated TC can harbour foci of PDTC and APC, strengthen this hypothesis that the progress is possible due to stepwise additions of molecular pathogenesis with increased mutational, copy number, and epigenetic events [66]. Mutations including *TERT* promoter, *TP53*, *EIF1AX*, and genes involved in the PIK3CA-AKT-mTOR pathway, *SW1/SNF* complex and mismatch repair genes are particularly involved in the more advanced TCs [67].

Progression from Follicular thyroid cells to papillary thyroid cancer (PTC) or follicular thyroid cancer (FTC) is marked by mutations activating the MAPK- or PI3K-AKT signalling pathways, illustrated by the arrows. Further progression to poorly differentiated thyroid cancer (PDTC) is characterised by additional mutations and rearrangements, augmenting both signalling pathways simultaneously. Anaplastic thyroid cancer (ATC) is characterised by further genetic events, especially in oncogenes like P53, as well as epigenetic alterations and infiltration of immune cells, creating an intracellular microclimate that favours genetic instability and oncogenesis.

Interestingly, simultaneous activation of MAPK and PI3K-AKT signalling pathways becomes more frequent as the grade of the tumour progresses [49]. Overactive PI3K-AKT and MAPK signalling pathways can activate the Wnt/β-catenin pathway known to regulate growth, proliferation as well as stem cell differentiation, as well as the HIF1α-pathway involved in angiogenesis and altered cell metabolism. Catenin Beta 1 (*CTNNB1*) is involved in cell adhesion and invasiveness, and HIF1, is a strong activator of vascular endothelial growth factor A, *VEGFA*. Both MAPK and PI3K-AKT signalling pathways can also stimulate FOXO- and NFκB pathways as previously described. Specific genetic alterations also present in the more advanced cancers includes *TP53* that induces genomic instability, *ALK* and epidermal growth factor receptor (*EGFR*) [49,67].

ATC harbours more copy number variations and epigenetic alterations than PDTC and PDTC more than DTC, which also supports the model of progressive oncogenesis. In conclusion, the progression from DTC to PDTC and further to ATC is hence an accumulation of genetic and epigenetic events that synergistically amplify their tumorigenicity [68] (Figure 2).

## 3. Molecular Marker for Diagnosis and Management of Thyroid Cancer in DTC

### 3.1. The Diagnostic and Therapeutic Challenge of Indeterminate Nodules

One major way this knowledge of the molecular biology of thyroid cancer is being applied to management is in the diagnosis of thyroid nodules. The introduction of the Bethesda System for Reporting Thyroid Cytopathology (BSRTC) in 2009 established a standardized reporting system of cytology results of thyroid fine needle aspiration (FNA) [69] (Table 1). It enabled users to reliably estimate the occurrence of malignancy in thyroid nodules for benign (BII) and malignant (BVI) Bethesda categories in 70 to 80% [70]. However, 20–30% remain indeterminate (BIII-BV) and risk of malignancy (ROM) demonstrate high variability ranging from 5–75%, the reasons for this are a matter of debate [69]. While intra- and interobserver variability is certainly one explanation, selection and publication bias need to be considered as well and result in a general overestimation of the ROM [69,71,72,73]. Moreover, the reclassification of the Encapsulated Follicular Variant of Papillary Thyroid Carcinoma (EFVPTC) to Non-invasive Follicular Thyroid Neoplasm with papillary-like nuclear Features (NIFTP) [74] not only established a new tumour classification [75] but as a consequence significantly lowered the rates of carcinomas in the indeterminate categories of the BSRTC [76]. Hence, predictions of ROM in the latest BSRTC classification differ from the previous version and, therefore impact decision making for the management of indeterminate nodules even more (Table 1).

Of note, malignancy of cytologically indeterminate nodules cannot be reliably diagnosed intraoperatively on fresh frozen sections due to low sensitivity and high false negative rates and increases cost and duration of operation [77,78]. In line with the 2015 ATA and the 2020 American Association of Endocrine Surgeons (AAES) guidelines, possible management strategies range from repeat FNA or surveillance to diagnostic surgery [15,17]. However, only 10–40% of indeterminate nodules submitted to surgery will be malignant on histopathology [79]. Consequently, molecular testing (MT) is considered as an adjunct to refine the cancer risk and to reduce the rate of diagnostic surgery. Furthermore, it may minimize the extent of the surgical procedure if no other indications for surgery are present (size, compressive symptoms, personal preference, high-risk features on imaging) [15,16,17,80]. However, in many health settings, commercially available MT is not easily available, and its cost can be prohibitive to use as will be discussed further (Table 2).

**Table 1:** The revised 2017 Bethesda System for Reporting Thyroid Cytopathology (TBSRTC) [81].

	Diagnostic Category	ROM ^1^ (NIFTP ^2^ Considered as Cancer (%))	ROM ^1^ (NIFTP ^2^ NOT Considered as Cancer (%))	Management Options
I.	**Nondiagnostic/unsatisfactory** (Cyst fluid, acellular specimen, other (e.g., obscuring blood, artefacts))	5–10	5–10	Correlate with clinical/radiological findings Consider repeat FNA ^3^
II.	**Benign** (Benign follicular nodule, Chronic lymphocytic (Hashimoto) thyroiditis, Granulomatous (subacute) thyroiditis)	0–3	0–3	Surveillance and US ^4^ follow up
III.	**Atypia of undetermined significance, *or* follicular lesion of undetermined significance**	6–18	10–30	Correlate with clinical/radiological findings Consider repeat FNA ^3^ Consider molecular testing
IV.	**Follicular neoplasm *OR* Follicular carcinoma** (specify if Hürtle cell features)	10–40	25–40	Consider molecular testing, Lobectomy
V.	**Suspicious for malignancy**	45–60	50–75	Total thyroidectomy or lobectomy
VI.	**Malignant** (Papillary thyroid carcinoma, Poorly differentiated thyroid carcinoma, Medullary thyroid carcinoma, Anaplastic thyroid carcinoma, Squamous cell carcinoma, Carcinoma with mixed features, Metastatic malignancy, Non-Hodgkin lymphoma, Other)	94–96	97–99	Total thyroidectomy or lobectomy

^1^ Risk of malignancy; ^2^ Noninvasive Follicular Thyroid Neoplasm with papillary-like nuclear features; ^3^ Fine needle aspiration; ^4^ Ultrasound.

### 3.2. Molecular Markers and Clinical Profiles in DTC

To enhance the diagnostic sensitivity of FNA results, enable molecular testing and to help guiding management in indeterminate nodules where no other indication for surgery (size, compressive symptoms, personal preference, high risk features on imaging) are present, molecular profiles and markers need to be identified and their diagnostic and prognostic role defined [87]. Molecular markers currently in use include immunohistochemistry, classical genomic alteration (point mutations, deletions, insertion), gene fusions causing rearrangements and translocation, copy number variations, microRNA (miRNA), and circulating markers of disease [17,88]. The most relevant alterations and markers will be discussed below.

#### 3.2.1. Genetic Markers

With around 80%, PTC is the most common entity of TC [89]. Despite excellent long-term survival, recurrence can cause significant morbidity and molecular markers can help to distinguish an aggressive from an indolent course [90]. Various driver mutations have been identified and associated with different histological subtypes [20]. However, PTCs can be classified by gene expression profiles to either *BRAF*-like (*BRAF*^V600E^, *RET*/PTC fusion, *NTRK* fusions) or *RAS*-like molecular subtypes (*RAS*, *BRAF*^K601E^, *PAX8/PPARG, PTEN*, *EIF1AX*) [20].

The most common mutation in 40–80% of PTC’s is *BRAF*^V600E^ [91]. Mainly seen in classic and tall cell PTC and less often in benign nodules, *BRAF*^V600E^ mutations are associated with a higher frequency of lymph node metastasis, extrathyroidal extension, recurrence, advanced clinical stage as well as poor response to radioiodine [92,93,94,95]. However, despite high specificity and positive predictive value for PTC, the absence of *BRAF*^V600E^ alterations alone harbour a low negative predictive value mainly due to the involvement of other driver mutations in the development of TC [96,97,98]. There have been conflicting findings on the prognostic value of *BRAF*^V600E^ [20,99,100] and only in combination with a *TERT* promotor mutation, it seems to be truly linked to aggressive PTC [101]. Due to this range of heterogenous phenotypes, *BRAF*^V600E^ is not an ideal exclusive marker to guide surgical management [102].

The rarer *BRAF*^K601E^ mutation is mainly associated with follicular variant PTC (FVPTC) but also benign adenoma. They demonstrated to show improved long-term outcome and only rarely cause disease related deaths [94,103]. However, *BRAF*^K601E^ mutation is not diagnostic for cancer due to a rather low positive predictive value (PPV) [104].

Despite being specific for PTC, *RET* rearrangements/fusion are identified only in about 7% of PTCs but frequencies vary deeply [20]. Almost exclusively found in PTC, the most common are the *RET/PTC1* and *RET/PTC3* accounting for about 90% of rearrangement in PTCs [20,105]. Some authors suggest that *RET/PTC1* relates to prognostically advantageous subtypes (more common in classic or diffuse sclerosing PTC), while *RET/PTC3* appears to occur in more aggressive phenotypes (solid variant PTC) [37,106,107]. Although, RET/PTC rearrangements seem to be associated with lymph node metastasis [94] and even multifocality [108] patients respond well to post-operative radioiodine (RAI) ablation and, therefore, follow a less aggressive course [109].

While the three *RAS* mutations (*HRAS*, *NRAS* and *KRAS*) play a smaller role in PTC (6–20%) [110], they are with 90% most common in FVPTC [111]. This subtype has been associated with infrequent spread to regional lymph nodes and good RAI avidity [20,112]. Interestingly, *RAS* mutations are, beside *PAX8/PPARG* and *THADA/IGF2BP3* gene fusions (22% of NIFTP) the predominant mutation in 67% of NIFTP [113,114] but also in a large number of benign follicular adenoma (20–25%) [21,115]. Therefore, *RAS* mutations alone cannot sufficiently predict malignancy but *RAS* mutated benign or borderline lesions represent precursor of malignancies [116] and may therefore necessitate surgical treatment such as lobectomy and close follow-up [74,117,118,119].

While the molecular landscape of PTC has been well described with current sequencing technologies, the molecular characterization of FTC still requires further clarification. The most common mutation in FTC are *RAS* and *PAX8/PPARG* fusion oncogenes, found in 27–50% [110] and 12–53% [21] respectively. However, additional genetic mutations, such as PTEN, PIK3CA, EIF1AX, DICER1, TSH Receptor, but also TERT promotor mutations are thought to be required to promote carcinogenesis and transformation of a follicular adenoma to FTC [21,120,121]. Although, some authors suggest an improved prognosis with *PAX8/PPARG* [122] rearrangement and worsened outcome with *RAS* mutation, only the total mutational burden seems to be an independent predictor of worse outcome, rather than single mutation alone [120,123,124]. Hürtle cell carcinomas (HCC), a variant of FTC, are genetically distinct with mainly CNAs and mutations in mitochondrial DNA or non-mitochondrial genes that interact with mitochondrial function. To date, none have been proven reliable [125]. Hence, proof of vascular invasion is still necessary to distinguish HCC from Hürtle cell adenoma (HCA).

#### 3.2.2. MiRNA Markers

Specific patterns of miRNA have been identified in large number of studies and are one of the novel methods to improve diagnosis in TC on FNA and also in “liquid biopsies” such as plasma samples due to their high stability in fluids and tissue specificity [31,126,127,128,129,130].

MiRNAs have been classified into a group of oncogenic and tumour suppressor miRNAs, which are based on their expression profile and their effect on cancer related signalling pathways [28,131,132]. The upregulation of oncogenic MiRNAs inhibits apoptosis and induce cell proliferation and growth, invasion and metastases by modulating target genes of several signalling pathways such as the PI3K/AKT/mTOR-, adipocytokine-, Hippo-, Wnt/β-catenin-signalling pathways [28]. In contrast, downregulation of tumour suppressor miRNAs results in loss of inhibition of cell proliferation and migration, and EMT in the MAPK, PI3K/AKT, NFκB or GSK-3β/β-catenin pathways [27,131,132,133,134]. Although miRNA have extensively been studied in PTC, distinct miRNA patterns were identified in different TC subtypes targeting different target genes [28]. Some examples of target genes in PTC are *Zinc Ring Finger 3 (ZNRF3)-*, cytokine receptor *KIT-* or the *CXCL12* gene [27,134,135].

Moreover, miRNAs have been successfully demonstrated to be able to discriminate benign from malignant lesions in PTC (e.g., miRNA-221, miRNA-222, miRNA-181b, miRNA-146b) and FTC/HCC (e.g., miRNA 148b–3p, miRNA-484) and could be a guide to avoid unnecessary surgery [27,136,137]. Moreover, certain miRNAs were also identified in APC, such as miRNA-19a [26]. However, panels of miRNAs may have a higher sensitivity and specificity than single miRNA in distinguishing thyroid nodules [127,138]. MiRNA profiling also showed that certain expression levels correlate with clinicopathological features (subtype, tumour size, multifocality, extrathyroidal extension and metastasis) as well as aggressiveness [27,32,139] and offer many opportunities for future research.

#### 3.2.3. Long Noncoding RNA Markers

Several specific lncRNA are dysregulated in TC in comparison to benign thyroid lesions and the presence of certain lncRNA have been associated with poorer TC prognosis [140,141,142,143]. LncRNA panels represent a promising novel adjunct method to distinguish malignant from benign thyroid lesions [144], however, not yet clinically available. An advantage with lncRNA markers is that they are tissue specific and present in circulating blood, enabling the possibility of a blood sample analysis to detect the presence of thyroid cancer in the future [145].

#### 3.2.4. Proteome Based Markers

Protein expression profiles (Proteomics) have been increasingly applied to search for novel diagnostic and prognostic marker [146]. They offer an unbiased platform to further investigate the whole proteome [147]. While several proteins have been identified (e.g., Prohibitin, ATP Synthase D chain, HSP70, PRDX, A100A6, TGFBI, E-Cadherin, A1AT) to be altered in malignant lesions, further preclinical and clinical study are required to assess their clinical applicability [53,146].

### 3.3. Molecular Testing (Table 2)

As demonstrated, mutational analysis for single genes has not adequately provided enough accuracy to properly guide decision making of thyroid nodules. The choice of MT is based on performance factors such as specificity, sensitivity, PPV, Negative predictive value (NPV), and prevalence. It should either provide the information if a test can “rule out” cancer (likelihood that a nodule is benign) and reduce diagnostic surgery for benign nodules or “rule in” cancer (likelihood that the nodule is malignant) to optimize extent of surgical treatment [82,117]. Therefore, high sensitivity and NPV would be ideal to “rule out” malignancy, while high specificity and high PPV would help to “rule in” malignancies [148].

Two MT techniques have been developed that avoid the use of single marker testing by using multiple markers to improve their performance, utilise *somatic mutation and rearrangement testing* and *gene expression or sequencing classifier (GEC, GSC)* [149]. *Somatic and mutation and rearrangement testing* initially started with the development of multigene panels such as the 7-gene panel (7GP) which included common genetic alterations (*BRAF*^V600E/*K601E*^, *N*-, *H*-, *K-RAS*, *PAX8/PPARG*, *RET/PTC* 1/3) [150]. The 7GP has been further extended and incorporated into 2 commercially available tests: The ThyGenX^®^ (7GP) + ThyraMIR^®^ (10 miRNA panel) [85] and ThyroSeq^®^ test. The ThyraGeNEXT/ThyraMIR^®^ is a further extension of the ThyGenX^®^ + ThyraMIR^®^ test and includes additional DNA and RNA, such as the prognostically relevant *TERT* mutation as well as specific alterations for Hürtle cell lesions. The first study of clinical performance reports that patients with Bethesda III and IV nodules with low risk mutations had high probability (94%) of remaining cancer-free (2 year follow-up), whereas moderate- and high-risk results demonstrated a probability of malignancy of 53% and 70% respectively [151]. This test might help to guide surgical planning for initial resection, rather than a 2-stage diagnostic lobectomy followed by definitive surgery as well as avoiding surgery for a benign disease [151]. The extent of surgery has not been addressed [85,151].

Following the initial ThyroSeq v0 (7GP) [150], the application of next generation sequencing (NGS) allowed to progress to the current version of the ThyroSeq v3, which tests for 112 genes, including copy number alterations (CNAs) [152,153]. As a “rule out” test with a benign call rate (proportion of FNA with negative MT as predictor of avoidable diagnostic surgery) of 61% of all Bethesda III and IV nodules and 82% of indeterminate nodules the ThyroSeq v3 could help to prevent diagnostic lobectomy [86,152]. However, further studies are needed to evaluate the clinical relevant performance [154].

The Affirma Gene Sequencing Classifier (GSC) [84] is the latest version of the mRNA-based Affirma Gene Expression Classifier (GSC), which initially consisted of a 167-gene microarray and 6 gene malignancy classifier panel and was a classical “rule out” test [155]. The Affirma GCS incorporated BRAF and RET/PTC and additional expression marker for Medullary thyroid cancer (MTC) and Hürtle cell lesions and improved specificity, while maintaining high sensitivity and NPV of the classical “rule out” GEC [84]. The GCS therefore remains a good tool but seems to be inferior to other tests in terms of benign call rate of 54% and 68% of histologically benign thyroid nodules classified as negative [84].

To date, no MT has been studied to determine the extent of surgery (lobectomy versus total thyroidectomy) under the 2015 ATA guidelines, which significantly modified the recommendation of surgical management of 1–4cm TCs [15]. Only multiple mutations, such as *BRAF^V600E^* with *TERT* promoter have been demonstrated to significantly impact PTC recurrence and mortality, and might therefore, be included in the decision making of surgical management, and offer the opportunity for further research [156].

### 3.4. Current Challenges and Limitations of Molecular Testing

There are several concerns regarding a wide implementation of molecular testing. First, validation studies of currently available MT were performed before the introduction of NIFTP and likely classified many NIFTP as malignant, which leads to altered predictive values of molecular tests. Hence, validation studies need refinement to appropriately address the impact of the NIFTP reclassification. Second, the identification of the appropriate clinical scenario, where MT might be useful, as well as the interpretation of results is challenging, and might result in over- or under-treatment [157]. Third, some studies showed, that only 7.9–8.4% of patients had altered surgical decision making as a result of molecular testing [158,159]. Fourth, MT has not been validated in paediatric patients [17]. Fifth, the correlation between presence of mutations and occurrence of malignancy is imprecise, since benign nodules harbour mutations, while some malignancies have none detectable with commonly used platforms [160]. Sixth, molecular tests are expensive, ranging from 1675–3500 USD [80], reports on cost effectiveness are conflicting, since expenses on MT might indeed be lower than diagnostic lobectomy [161] and are dependent on local health care settings. However, the impact of long-term surveillance on cost of conservatively managed patients need further work-up. Seventh, MT is not available worldwide and delays of treatment need to be considered and weighed up against the benefits. Finally, molecular marker negative patients lack histological confirmation on a surgical specimen and final diagnosis cannot be established. While many issues are yet to be resolved, MT is widely used in some health care settings and long-term follow up of these patients and further research will hopefully further clarify the benefit of MT in the management of thyroid nodules and thyroid cancer.

## 4. Molecular Marker for Surveillance in DTC

One molecular marker that is widely used for surveillance is serum thyroglobulin (Tg) level, which reflects remaining benign or malignant thyroid tissue. Tg achieves the highest sensitivity and specificity when used for surveillance with very low serum levels in patients who underwent total thyroidectomy, removal of involved lymph nodes and radioiodine ablation [162]. While Tg levels should be measured every 6–12 month during initial follow-up for all DTC patients, the timing of further Tg measurements depend on ATA risk stratification and recommendations vary [15]. Of note, serum Tg levels are influenced by the degree of thyroid stimulating hormone (TSH) stimulation [15]. A further limitation of measuring Tg is the presence of Thyroglobulin antibodies (TgAb), which is detected in 20% of TC patients and causes unreliable and false negative Tg results [163]. A recent meta-analysis demonstrated that the risk of developing lymph node metastasis and cancer persistence/recurrence was two times, respectively three times, higher in TgAb positive patients [164,165]. Moreover, TgAb persistence or increase leads to a 10 fold higher risk of cancer persistence/recurrence and 15 fold increase in cancer mortality, especially in patients who showed a post-treatment decrease of <50% [164]. Therefore, TgAbs should be assessed along Tg during follow up [15] and a rise should set off further investigations independently of Tg levels [15].

Another promising approach is the use of miRNA as predictor of early relapse. These patients showed a decline of circulating miRNA 146b-5p, miRNA-221-3p, miRNA-222-3p and miRNA-146a-5p after surgical resection [32,166]. Moreover, serum levels of miRNA-221-3p and 146b-5p increased after 2-year follow-up of PTC with evidence of structural recurrence, even in Thyroglobulin negative assays [166]. The use of circulating miRNA and other circulating biomarkers faces several challenges and further studies are required to address the application in TC diagnosis and surveillance [167].

## 5. Molecular Markers to Guide Targeted Treatment for Advanced TC

### 5.1. Diagnosis of Advanced TC, PDTC, and ATC

Patients with rapidly growing lesions in the thyroid require prompt investigation with imaging and biopsy. Molecular markers offer the possibility to further aid management of advanced TC. Advanced TC is defined upon its clinical aggressiveness, while the definition of PDTC and ATC require histological evaluation. PDTC is defined histologically by either the Turin or the MSKCC criteria. The Turin criteria defines a solid, insular or trabecular growth pattern, absence of nuclear features of PTC and one out of three; convoluted nuclei, necrosis or increased mitosis (>3 per 10hpf) [168]. The MSKCC criteria identifies presence of follicular cell differentiation with presence of >5 mitosis/10HPF and/or necrosis [169]. PDTC and ATC often present with infiltrative growth and vascular invasion. The histopathological characteristics of ATC are spindle-shaped, epithelioid or giant cells with marked cytological pleomorphism and extensive necrosis. PDTC often still express some TTF-1 and Tg, reflecting their origin from thyroid epithelium, while ATCs often have completely lost expression of theses markers [67]. ATCs are in most cases infiltrated by immune cells; neutrophils, T-cells and tumour-associated macrophages, promoting an inflammatory and oncogenic microenvironment [67,170].

Kunstman et al. published the first whole exome sequencing of an ATC cohort showing distinct patterns of high frequency mutations in *BRAF*, *P53* and *RAS* [171]. In addition, Landa et al. demonstrated a high *TERT* promoter mutation both in PDTC and ATC, as well as mutated targets in the PI3K/AKT signalling pathway; *PIK3CA*, *mTOR*, *PTEN*; transcriptions factors *EIF1AX*, *NF1*; cell cycle regulators *CDKN1B*; histone methyltransferases (*HDAC*, *KMT*) and increased CNA and *ALK* involved fusion-oncogenes [25]. Specifically, for ATC is the increased mutational burden in combination of epigenetic alterations enhancing the effect of genetic mutations. Co-existence of both constitutive MAPK- and PI3K/AKT signalling also augments genetic instability. Pozdeyev et al. demonstrated a pattern of three gene mutational clusters of ATC, suggesting the ATC tumours were derived from either PTC (*BRAF*-like), HCC or FTC (*RAS*-like) [172]. ATC are complex in their genetic and epigenetic signature, however, some ATC retain their dependence of its driver mutation [173,174].

### 5.2. Impairment of Iodine-Handling Machinery in Advanced Thyroid Cancer

The three cornerstones of treatment in DTC is surgery, adjuvant RAI-ablation, and TSH suppression. However, in PDTC and ATC these cornerstones are challenged with tumour biology promotes vascular and extrathyroidal invasion, downregulation of the expression of the TSH receptor gene and genes involved in the iodine-handling machinery, reducing RAI efficacy. Additional treatments with external beam radiation (EBR) and chemotherapy with taxanes in combination with carboplatin or doxorubicin can be useful in reducing tumour- and metastasis size and can sometimes enable debulking surgery to avoid airway obstruction and achieve local tumour control; however, response rates to chemotherapy are low, thereby limiting their use [175]. The prognosis for patients with advanced thyroid tumours with distant metastasis upon presentation is poor, around 60%, for 10-year survival and only 10% for RAI-refractory TC in comparison to the excellent prognosis of 95% for DTC making a better understanding of biology a strong impetus to improve outcomes for these patients [176,177].

The main and unique function of follicular cells is to use iodine to synthesise thyroid hormone. Iodide is transported into the cell by NIS which is the sodium-iodine symporter located in the basal membrane. At the apical membrane, pendrin transports iodine out of the cell into the lumen of the thyroid follicle. In the lumen, iodine undergoes oxidation by TPO and is incorporated into tyrosine residues of Tg, which is later cleaved through proteolysis to produce thyroid hormones, thyroxine (T4) and triiodothyronine (T3). This machinery is upregulated by TSH activation of the TSH-receptor (TSHR) and exploited in RAI-ablation and requires to be intact for RAI treatment to be effective [178].

*BRAF*^V600E^ leads to decreased or inhibited expression of *NIS*, *TSHR*, *TPO*, *TG* as well as *SLC26A4*, the gene, which encodes for pendrin [34], which can lead to inability for the TC to incorporate iodine. Consequently, this causes TC RAI-refractory disease (RAIRD), and DTC with *BRAF*^V600E^ mutations are significantly more likely to be RAI refractory [179] and, to a high extent, PDTC and especially ATC, are RAIRD. Additional genes, like *PAX8* has also been shown to be a potent repressor of NIS in thyroid cells [180]. RAIRD is associated with FDG-PET avidity, which can therefore be used for diagnosis. RAIRD is clinically established when patients, appropriately TSH-stimulated, fail or loose ability to concentrate iodine in metastases or if there is tumour progression despite significant RAI uptake [181]. In these patients, there is no further role for RAI if their iodine refractory status cannot be reversed.

### 5.3. Tyrosine Kinase Inhibitors

**Figure 3:**
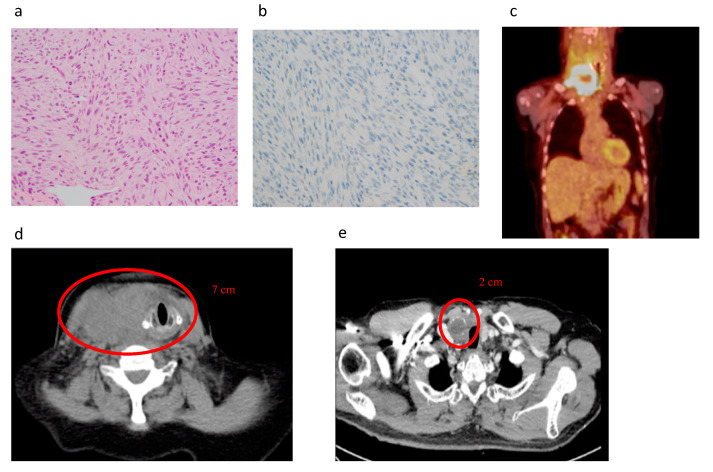
Histopathology and images of case report described in Box 1. (**a**) Haematoxylin and eosin stain of tumour, (**b**) *BRAF^V600E^* stain of tumour, (**c**) PET scan at initial presentation, (**d**) CT at initial presentation, (**e**) CT after one month of daily Lenvatinib 20mg treatment and 50Gy external beam radiation.

The increased knowledge of the molecular biology of PDTC and ATC has enabled targeted treatments of the affected signalling pathways. Inhibitors of intracellular tyrosine kinase signalling pathways, tyrosine kinase inhibitors (TKIs), have shed new hope for the treatment of advanced TC. Although they do not cure, they can slow down growth, reverse adverse effects of the mutations, when traditional treatments are ineffective. TKIs have also shown results in reversing the TC oncogenesis, enabling for the traditional treatments to again become effective. This effect is demonstrated in the case report (Box 1, Figure 3) which shows how use of TKIs in a neoadjuvant setting can achieve local control and effective palliation for an aggressive advanced TC.

The first clinically approved TKIs for RAIRD advanced TC were Lenvatinib and Sorafenib, which are both orally available multi-tyrosine kinase inhibitors. Lenvatinib inhibits vascular endothelial growth factor receptor 1-3 (VEGFR1-3), Fibroblast growth factor 1-4, RET, c-KIT and platelet derived growth factor receptor beta (PDGFRβ) [182]. The main tumour reducing effect is likely the anti-angiogenetic effect, but secondary effects of inhibiting additional kinases including RET and FGFR may have additional effects [183]. Sorafenib inhibits RAF, VEGFR 1-3, PDGF receptor and RET [184]. The SELECT trial, a major phase III study compared Lenvatinib to placebo in patients with RAIRD and showed a disease free survival of 18.3 months in the treatment arm compared to 3.6 months in the placebo group [185] which established the use of Lenvatinib as first line treatment of progressive systemic RAIRD. The DECISION study, a multicentre, randomised, double-blind phase III trial, comparing Sorafenib to placebo, showed progression-free survival of 10.5 months compared to 5.8 months with placebo [186]. TKIs usually require long-term use but diarrhoea, fatigue, hypertension and hand-foot skin reactions are frequent side effects [181] and in the SELECT trial, 14% of the patients treated with Lenvatinib have to cease treatment for this reason.

Since the entry of Lenvatinib and Sorafenib, there are several clinical trials evaluating effects of targeted TKI, tailored for a specific driver mutation. The *BRAF*^V600E^ inhibitor Vemurafenib [187] and Dabrafenib are clinically approved for *BRAF*^V600E^ positive RAIRD TC. Liu et al. in 2007 showed that by inhibiting MEK in TC cell cultures, the expression of genes involved in the iodine handling machinery could be restored [188] and the clinical available MEK inhibitor Selumetinib was shown to restore the NIS expression in TC [189]. In a study by Iravani et al. [190] with RAIRD, patients with either *NRAS* or *BRAF*^V600E^ were treated with MEK inhibitor (Trametinib) and BRAF- and MEK inhibitors (Dabrafenib and Trametinib or Vemurafenib and Cobimetinib) for four weeks, showing that all three *BRAF*^V600E^-mutated patients and one *NRAS* patient demonstrated restoration of RAI uptake. Recently, the BRAF inhibitor Dabrafenib and the MEK inhibitor Trametinib was also approved for treatment in ATC, in patients not responding to locoregional treatment options [191]. Today there are several other available targeted TKIs; NTRK inhibitor Larotrectinib [192], mTOR inhibitor Everolimus [193], ALK inhibitor Crizotinib [194] and the RET inhibitor Selpercatinib was recently clinically approved for RAIRD metastatic advanced TC with RET-fusion genes [195,196]

Box 1 **Case report.**

A man in his 60s presented to our clinic with one-month history of rapidly enlarging right neck mass and voice change. He had a family history of thyroid cancer. A core biopsy was performed that showed spindle cell type ATC which was *BRAF*^V600E^ negative (Figure 3a,b). Laryngoscopy showed a right recurrent laryngeal nerve (RLN) palsy. FDG-PET scan was performed showing high uptake in the lesion on the neck as well as pulmonary and bone metastasis (Figure 3c). On imaging and clinical exam, the cancer was thought to unable to be resected without significant morbidity due to local invasion (Figure 3d). He was started on Lenvatinib and received external beam radiation to the neck and showed a response with regression of tumour from 7 to 3 cm within one month (Figure 3e). He developed side effects of Lenvatinib with ulcerations of feet and diarrhoea and due to the reduction in size of the cancer underwent a thyroidectomy where the Right RLN was invaded in the tumour and sacrificed. Histopathology showed a *BRAF*^V600E^ negative, *ALK* negative, *P53* positive in >50% of tumour. Postoperatively he was then restarted on lower dose Lenvatinib and three months later started a combination with Pembrolizumab due to the development of liver metastasis. He achieved good local control in his neck but unfortunately developed pulmonary infection and died 18 months following his initial surgery.

While many trials of TKIs were mutation agnostic it is possible identification of the driver mutation could improve response of advanced TC with TKIs. The advances of hybridisation capture-based NGS panels, Memorial Sloan Kettering integrated mutation profiling of actionable cancer targets (MSK-IMPACT), and Foundation One, which can detect somatic and germline mutations, are becoming more accessible for clinicians to customise treatment for patients with advanced TC [197,198].

### 5.4. Application of Molecular Markers to Decision Making for Advanced TC

The decision of when to start TKIs for advanced TC can be difficult with Tg doubling time, radiological progression or unresectable tumours indications to consider systemic treatment. As local, structural recurrence can be treated with surgery, EBR, radiofrequency-, cryo-, or alcohol ablation, it is suggested that TKIs should be reserved for radio-refractory patients with rapid tumour progression and severe symptoms that threaten life [15]. In addition, the use of TKIs have value in neo-adjuvant treatment in patients with advanced TC to reduce tumour size to enable complete macroscopic resection (R1), for symptomatic relief and also for enabling further RAI ablation [190,199].

How we apply this knowledge of biology to the clinic and choose which TKI to use initially is the subject of further assessment and research. While initial trials of *Lenvatinib* [185] and *Sorafenib* [186] did not suggest a difference in response according to driver mutation for the treatment of metastatic disease RAIRD, subsequent reports have suggested that redifferentiation may be higher in patients with RAS mutated compared to *BRAF*^V600E^ mutated cancers [200].

In the setting of ATC, there is evidence of response specific to mutations such as the MEK inhibitor for *BRAF*^V600E^ mutated cancers [191] and response to *Lenvatinib* for RAS mutated cancers [201] is an area of active research. In our experience, patients with *BRAF*^V600E^ WT ATC have responded to *Lenvatinib* as shown in our case report (Box 1) and achieved remarkable responses allowing some ATCs to be down staged and proceed to surgery to achieve local control and effective palliation (Box 1, Figure 3). In addition, the availability of targeted therapies for rarer drivers such as NTRK inhibition with *Larotrectinib* [192], mTOR inhibition with *Everolimus* [193], ALK inhibition with *Crizotinib* [194] and the RET inhibition with *Selpercatinib* mean it is important to assess patients tumours with advanced disease for the genetic driver mutation.

**Figure 4:**
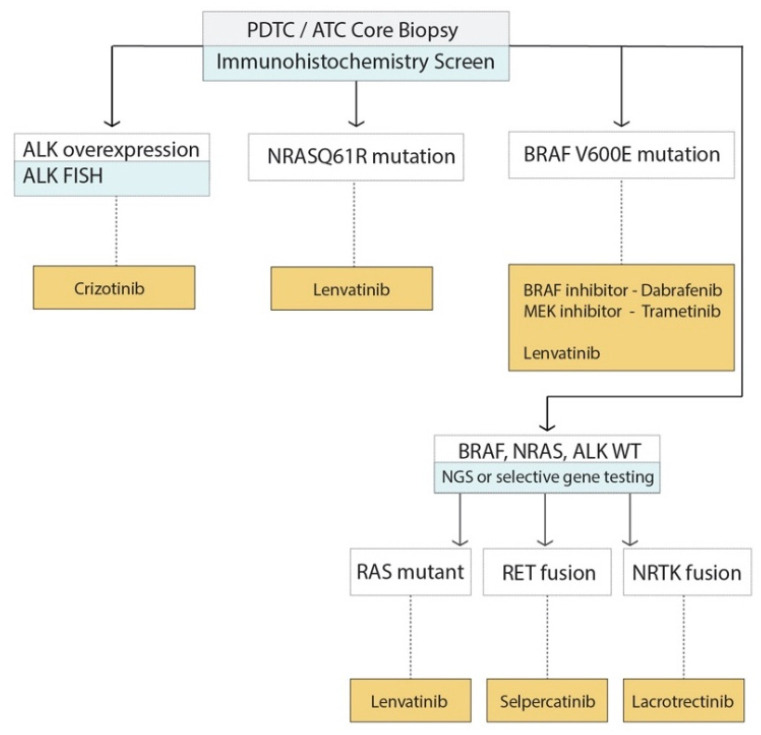
Suggested flow chart of use of molecular testing in advanced thyroid cancer.

Sequencing of all advanced TC would be of great value for increased understanding of the underlying driving mutations, modifying mutations, and to individually tailored targeted treatments; however, currently, it is too costly and the time taken for sequencing results (with a usual turnaround of 6–8 weeks) makes this approach not feasible for many endocrine centres and for patients presenting with advanced disease. One possible approach is to triage advanced tumours for further mutation testing using immunohistochemistry (IHC) which is the practice of our institution (Figure 4). In this approach, *BRAF*^V600E^ mutation specific IHC, *RASQ61R* mutation specific IHC and ALK IHC is performed. For patients with high ALK expression, this is used as a surrogate for *ALK* gene rearrangement, which is then confirmed by *ALK* Fluorescence in situ hybridization (FISH). For patients who are *BRAF* wild type, *RASQ61R* negative and with normal ALK expression further genetic testing is performed using targeted next generation sequencing targeted DNA and RNA sequencing by NGS (Figure 4). An RNA panel is used to detects transcripts from 76 fusion variants from eight genes and include *RET*, *ALK* and *NTRK3, BRAF, PPARG, THADA*, and *MAML2*. Meanwhile, the DNA panel targets 2023 mutations in 14 key cancer genes and include *AKT1, BRAF, CTNNB1, GNAS, HRAS, KRAS, NRAS, PIK3CA, PTEN, RET, TERT, PT53*, and *TSHR*. Depending on the results, this information is used to consider the choice of targeted therapy (Figure 4).

Molecular markers can be utilized to type advanced TC and identify the driving or modifying mutation, which can then be useful for choice of targeted treatment. This flowchart summarizes the management of molecular testing at our clinic and the selection of currently available therapies.

## 6. Conclusions

Molecular markers for diagnosis, prognosis, surveillance and treatment are becoming more important as knowledge of the molecular mechanisms of thyroid cancer increases. The potential of molecular testing in DTC is immense in terms of diagnosing cancer, assess prognosis and ultimately avoid surgical overtreatment. However, molecular testing still faces several challenges, which needs be addressed before a broad implementation into clinical practice can be achieved. There are currently several ongoing clinical trials, which evaluate new, targeted treatments for advanced thyroid cancers, where the prognosis was previously poor. This will allow clinicians to work with a larger arsenal of diagnostic and therapeutic tools to combat differentiated and advanced thyroid cancer. The key to success is molecular testing and personalised medicine, to obtain flexibility to tailor treatment for each unique cancer type and patient.

## Figures and Tables

**Table 2:** Summary of recent commercially available molecular tests [17,82,83].

	Affirma^®^ GSC	ThyGenX/ThyraMIR^®^ ThyraGeNEXT/ThyraMIR^®^, *	Thyroseq v3^®^
Methodology	mRNA gene sequencing and gene expression	NGS and mRNA sequencing, polymerase chain reaction miRNA expression	NGS of DNA and mRNA
Tested alterations	511 mRNA panel, gene expression of 1115 genes, CAN ^3^	7–10 DNA and mRNA panel, 10 miRNA	112 DNA and mRNA panel (>12,000 variants and 150 gene fusions), CAN ^3^, gene expression
Ideally applicable for	Bethesda III/IV, Hürtle cell lesions	Bethesda III/IV/V	Bethesda III/IV/V, Hürtle Cell lesions
Report	GSC results as benign *or* suspicious *BRAF^V600E^*, medullary TC, *RET/PTC 1*/*3* specified *TERT* not offered	Reports if oncogene in THyGenX/ThyraGeNEXT present If ThyGenX/ThyGeNExt negative, then ThyraMIR reported as high or low risk *BRAF^V600^, RET/PTC, TERT* mutations specified	Positive or negative with detailed result for each marker *TERT* and *TP53* offered
Interpretation of test results	Benign: consider surveillance Suspicious: consider surgery	ThyGenX/ThyraGeNEXT positive: consider surgery ThyraMIR high risk: consider surgery ThyraMIR low risk: consider surveillance	Negative: consider surveillance Positive: consider surgery
Validation Study	Patel et al. [84]	Labourier et al. [85]	Steward et al. [86]
Nodules examined	*n* = 190	*n* = 109	*n* = 247
Prevalence of cancer	24%	32%	28%
Sensitivity	91% (79–98)	89% (73–97)	94% (85–100)
Specificity	68% (60–67)	85% (75–92)	82% (63–84)
NPV ^1^	96% (90–99)	94% (85–98)	97% (93–99)
PPV ^2^	47% (36–58)	74% (58–86)	66% (56–75)
	“Rule out”	“Rule in” and “Rule out”	“Rule in” and “Rule out”

^1^ Negative predictive value; ^2^ Positive predictive value; ^3^ Copy number alterations; * The ThyGeNEXT expanded DNA and mRNA panel among them *TERT, PIK3CA, ALK, PTEN* mutation.
